# Potential of tadalafil and tadalafil-cellulose nanocomposite in preventing postsurgical abdominal adhesions in a rat cecal abrasion model

**DOI:** 10.1038/s41598-025-14894-0

**Published:** 2025-08-25

**Authors:** Ahmed Abdelrahiem Sadek, Mahmoud S. Sabra, Marwa F. Ali, Hani Nasser Abdelhamid, Kamal Hussein

**Affiliations:** 1 Department of Surgery, Anesthesiology and Radiology, Faculty of Veterinary Medicine, Assiut University, Assiut, 71516 Egypt; 2 Department of Pharmacology, Faculty of Veterinary Medicine, Assiut University, Assiut, 71516 Egypt; 3 Department of Pathology, Faculty of Veterinary Medicine, Assiut University, Assiut, 71516 Egypt; 4 Department of Chemistry, College of Science, Imam Mohammad Ibn Saud Islamic University (IMSIU), 11623 Riyadh, Saudi Arabia; 5 Tissue Culture and Stem Cells Unit, Molecular Biology Researches and Studies Institute, Assiut University, Assiut, 71526 Egypt

**Keywords:** Intra-abdominal, Adhesions, Tadalafil, Cellulose, Anti-adhesion, Postsurgical, Preclinical research, Biomaterials

## Abstract

The formation of postoperative intra-abdominal adhesions is a significant challenge in veterinary practice worldwide. Thus, several attempts have been made to identify agents that prevent the occurrence of these postsurgical adhesions. However, finding an ideal and effective agent remains a challenge. Herein, we investigate the potential of tadalafil and tadalafil/cellulose composite as promising therapeutics for preventing postsurgical intra-abdominal adhesions. A cecal abrasion model was established in 30 rats, which either left untreated or treated with tadalafil, cellulose, or tadalafil/cellulose. After 2 weeks, the adhesion formation was evaluated based on gross appearance, oxidative stress markers, pro-inflammatory cytokines, histopathological analysis, and immunohistochemical staining. Compared to the adhesion group, gross and histopathological findings revealed that both the tadalafil and cellulose groups significantly decreased adhesion formation, with better results observed after tadalafil treatment. Importantly the tadalafil/cellulose treatment completely prevented adhesion formation. Additionally, the treated groups showed reduced levels of malondialdehyde (MDA), tumor necrosis factor-alpha (TNF-α), and interleukin-6 (IL-6), while increasing the level of reduced glutathione (GSH) compared to the adhesion group. Furthermore, the treated groups reduced the expression of macrophage markers. These findings suggest that the intra-abdominal application of tadalafil and tadalafil/cellulose following abdominal surgery holds promise as a clinical strategy to prevent postsurgical intra-abdominal adhesions, with tadalafil/cellulose demonstrating superior efficacy.

## Introduction

Abdominal surgery is one of the most common procedures in veterinary practice, including ovariohysterectomy, caesarean section, abdominal organ biopsy as well as surgeries involving gastrointestinal tract, urinary system, displaced abomasum, and equine colic^1–5^. A common complication following intra-abdominal surgical interventions is the formation of adhesions, which result from excessive collagen deposition at the injured site due to improper tissue repair^6,7^. These peritoneal adhesions present an important clinical challenge with economic drawbacks due to the development of various complications, including abdominal pain, organ dysfunction (e.g., bowel obstruction or infertility), and increased morbidity and mortality^7–9^. The key factor in the mechanism of formation of such postsurgical peritoneal adhesions is the dysregulation that occurred during the natural process of tissue repair between the deposition of fibrin on the serosa of the injured tissue and the fibrinolysis process^7,10^. After surgery, tissue trauma triggers local hypoxia, initiating a cascade of events, including coagulation, inflammatory reactions, inhibition of fibrinolysis, fibroblast infiltration, collagen deposition, fibrin matrix organization, and tissue remodeling^7,9–11^.

The prevention or reduction of the risk of intra-abdominal adhesion formation following surgery primarily depends on two approaches: adopting meticulous surgical procedures and application of adhesion-inhibitory adjuvants^7,11^. Adhesion-inhibitory adjuvants are materials designed to reduce or prevent the formation of the postsurgical intra-abdominal adhesion bands by interfering with the adhesion formation process. These adjuvants may act as a barrier that prevents direct contact between traumatized serosal surfaces and the peritoneum and/or adjacent tissues^7,9–11^. Among the diverse anti-adhesive products, chitosan^12^ mitomycin C^13^ cellulose^14^ gelatin^15^ hyaluronic acid^16^ heparin^17^ phlorotannin^18^ and prednisolone^19^ were evaluated, even though a satisfactory ideal material still missing.

Tadalafil, a polycyclic drug that belongs to the phosphodiesterase-5 (PDE-5) inhibitors, has gained significant attention in medicine over the past decades. It is a specific PDE-5 inhibitor with a prolonged duration of action^20^. It has been reported that tadalafil possesses antioxidant and anti-inflammatory properties^21,22^. Moreover, tadalafil has been used in the treatment of various conditions including pulmonary arterial hypertension^23^ male erectile dysfunction^24^ prostatic hyperplasia^25^ cisplatin-induced testicular toxicity^26^ chronic renal failure^27^ dexamethasone-induced gastric ulcer^21^ carbon tetrachloride-induced liver failure^22^ and diabetes mellitus^28^.

Recently biopolymers have been extensively integrated into medical applications^29,30^. Cellulose, a biocompatible and biodegradable carbohydrate homopolymer, is known for its minimal tissue toxicity. It is characterized by a unique physical and chemical configuration, including large surface area, excellent mechanical strength, adequate stability, and adjustable porosity^31–33^. Consequently, cellulose can be used alone or conjugated with other biomaterials for various medical applications, such as tissue engineering^34^ wound dressing^35^ and drug delivery^36^. Furthermore, cellulose has demonstrated efficacy in reducing the formation of intra-abdominal postsurgical adhesions, either alone or combined with other materials such as heparin, sodium hyaluronate, chitosan, and mitomycin C^9,13,37^.

Thus, the main objective of this study is to develop a biodegradable and effective product to prevent the formation of intra-abdominal adhesions after abdominal surgery by evaluating the ability of tadalafil and tadalafil/cellulose to diminish or prevent the formation of such adhesions. Moreover, it aims to assess the biocompatibility of the designed material *in vitro* and *in vivo*.

## Materials and methods

**Fig. 1:**
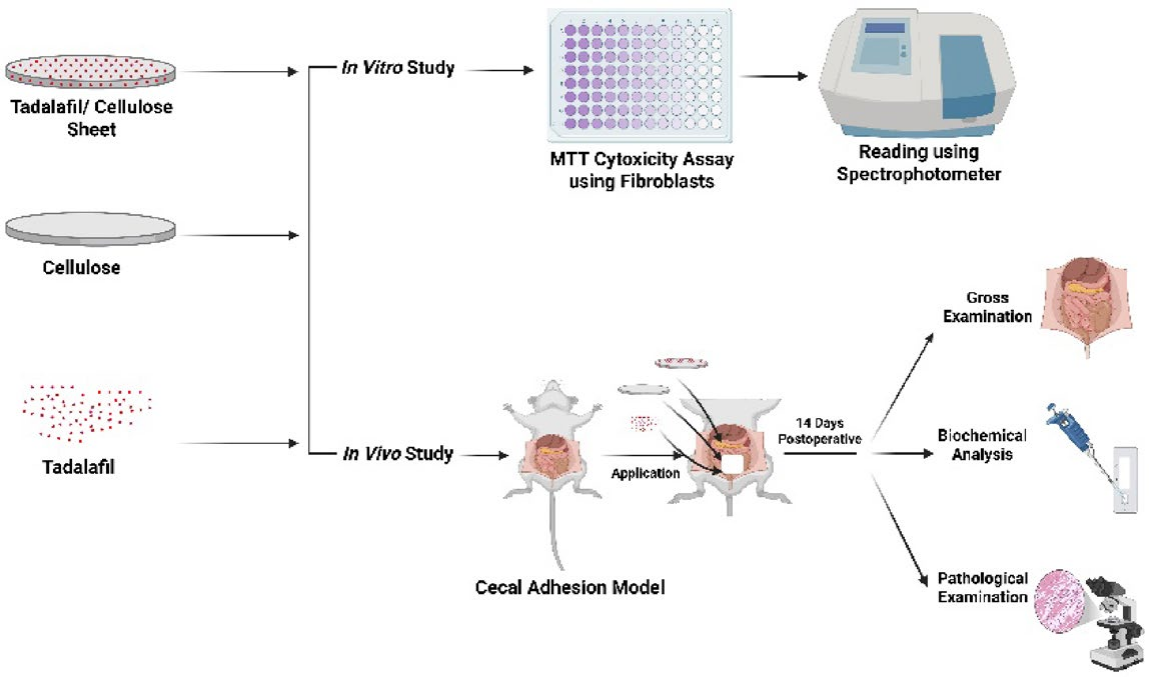
Graphical summary illustrating the overall study design, including material synthesis and characterization, *in vitro *cytotoxicity testing, and *in vivo* evaluation in a rat model of intra-abdominal adhesions (Created in BioRender, https://BioRender.com/7lp96sr).

As summarized in Fig. 1, this study involved three main stages; synthesis and characterization of the materials, *in vitro* cytotoxicity evaluation, and *in vivo* assessment of the materials’ potential to reduce or prevent the intra-abdominal adhesion formation in a rat cecal abrasion model.

### Ethical statement

All the experimental protocols in the present study received approval from the Ethical Committee of the Faculty of Veterinary Medicine, Assiut University (Approval Number: 06/2025/0311). All methods were performed in compliance with the Animal Research: Reporting of In Vivo Experiments (ARRIVE) guidelines. Animal handling and experimental procedures adhered to Egyptian regulations and the animal welfare standards outlined by the World Organization for Animal Health (OIE). All methods were performed in accordance with the relevant guidelines and regulations.

### Materials

#### Tadalafil/cellulose composite fabrication and characterization

Cellulose dispersion was prepared using Whatman® filter paper as a cellulose source via Ultrux at 15,000 rpm till dispersion. Tadalafil/cellulose composite was prepared as a thin film by casting it at room temperature. Typically, 20 mg of tadalafil powder (AK Scientific, Inc., USA) was dispersed in 100 mL cellulose fiber (1 wt%) via ultrasonication. Cellulose without the drug was also prepared following the same recipe.

The prepared material was characterized using the powder X-ray diffraction (XRD) patterns (Philips PW1700 diffractometer, Eindhoven, Netherlands) with a Cu Kα radiation diffractometer.

#### *In vitro* assessment of cytotoxicity

The cytotoxicity was evaluated using the [3-(4,5-dimethylthiazol)-2-yl]-2,5- diphenyltetrazolium bromide (MTT) assay. Mouse embryonic fibroblast (MEF) was maintained in the growth medium DMEM supplemented with 10% fetal bovine serum (FBS) and penicillin/streptomycin (1%) in a CO2 incubator at 37 °C. MEFs were seeded in 96-well plates and incubated for 24 h at 37 °C in a humidified atmosphere with 5% CO₂. After incubation, the culture medium was replaced with conditioned media prepared by agitating cellulose (4 µg/mL) in media at 37 °C and 100 rpm for 48 h, tadalafil solution (10 µM, 4 µg/mL), or a combination of both. Cells cultured in complete medium without treatment served as the negative control, while cells treated with 20% dimethyl sulfoxide (DMSO) were used as a positive toxic control. At 48 h, the medium was discarded and replaced with 5 mg/mL MTT solution, followed by incubation for 3 h. The MTT solution was then removed, and 0.1 mL DMSO was added to dissolve the formazan product. The absorbance was measured at 570 nm using a microplate reader. Cytotoxicity results were expressed as a percentage relative to the negative control group, which was set as 100%. All experiments were performed in triplicate.

#### *In vivo* study design

Thirty Wistar rats were used to evaluate the efficiency of tadalafil and tadalafil/cellulose nanocomposite in preventing the occurrence of postsurgical intra-abdominal adhesions. The apparently healthy adult female rats (8-weeks-old, 230 ± 20 g) were supplied by the Experimental Animal Unit at Department of Pathology, Faculty of Veterinary Medicine, Assuit University. Each rat was separately housed in a well-conditioned cage at the Veterinary Teaching Hospital, Faculty of Veterinary Medicine, Assuit University, with continuous unrestricted access to standard rat chow and water. Rats were adapted for 10 days in their new houses prior to further surgical intervention. They were assigned randomly into five equal groups (*n* = 6 per group) including the adhesion group, the tadalafil-treated group, the cellulose-treated group, the tadalafil/cellulose-treated group, and the sham group.

### Surgical induction of intra-abdominal adhesions in a rat model

The cecal abrasion model was selected in this study as a model for postsurgical intra-abdominal adhesions formation^38^. Surgical procedures were conducted under general anesthesia using isoflurane (Isoflurane AIT®, Arab Caps, Alexandria, Egypt). Anesthesia was induced in rats using an induction chamber (2–3.5% isoflurane in 100% oxygen) and maintained via a non-rebreathing system using a nose cone (1.5–3.5% isoflurane in 100% oxygen). The animals were then positioned in dorsal recumbency, and the ventral abdominal wall underwent strict aseptic preparation, including shaving, disinfection, and draping.

For surgical access to the cecum, a median celiotomy was performed at the umbilical region passing through the skin, abdominal muscles, and peritoneum. The cecum was gently exteriorized and subjected to wall scrubbing by a piece of dry sterile gauze to induce cecal abrasions, as indicated by the appearance of punctate hemorrhage. In the adhesion group, the abraded cecum was gently repositioned into the abdominal cavity without further intervention. In the tadalafil-treated group, tadalafil powder (20 mg/rat, AK Scientific, Inc., USA)^39^ was topically applied to the abraded cecal wall before relocation into the abdomen. In the cellulose- and tadalafil/cellulose-treated groups, the abraded cecum was carefully repositioned in its normal abdominal location, followed by intra-abdominal application of either a cellulose sheet (3 × 1 cm^2^) or a tadalafil/cellulose sheet (3 × 1 cm^2^) on these ceci, respectively. Lastly, the abdominal muscles and skin were sutured in a routine manner. In sham- operated rats, celiotomy was performed, followed by abdominal closure without any further surgical manipulation of the cecum.

Finally, the animals were returned to their individual cages with careful observation during the recovery period. The condition of the rats was checked daily for 2 weeks after surgery for any health or behavioral alterations. After this period, rats were sacrificed through cervical dislocation under isoflurane anesthesia for morpho-histological assessment of intra-abdominal adhesion formation.

### Gross assessment

After euthanasia of animals, a paramedian celiotomy was carried out, extending from the xyphoid cartilage to the pubis, to allow thorough examination of intra-abdominal adhesions between the cecum and the abdominal wall, other abdominal organs, or both. Adhesions were evaluated and scored semi-quantitatively, as previously described by Ibrahim et al.^38^ (Table 1).

**Table 1:** The criteria for the *in vivo* adhesion scoring according to Ibrahim et al.^38^.

Gross abdominal adhesion	
Scale	Adhesion
0	No adhesion
1	One adhesive band either between the organs or between the organs and abdominal wall
2	Two adhesive bands either between the organs or between the organs and abdominal wall
3	More than two adhesive bands between the organs or between the organs and abdominal wall or adhesions of the whole intestinal tract without the abdominal wall
4	Adhesion of the viscera directly to the abdominal wall

### Oxidative stress indicators analysis

At 2 weeks post-surgery, serum samples were collected for assessment of malondialdehyde (MDA) and reduced glutathione (GSH) levels. These biomarkers were analyzed spectrophotometrically using commercially available reagents from Schiffgraben, Hannover, Germany^40,41^.

### Tumor necrosis factor alpha (TNF-α) level Estimation

Commercial enzyme-linked immunosorbent assay kits (ELISA, Sunlong Biotechnology, Hangzhou, Zhejiang, China) were employed to quantitatively measure TNF-α levels in rat serum, strictly adhering to the manufacturer’s protocol. The procedure involved adding rat serum samples and TNF-α standards to 96-well plates precoated with monoclonal antibodies. Biotinylated detection antibodies specific to TNF-α were subsequently incorporated. The reaction was visualized by adding a chromogenic substrate, which caused a color change from blue to yellow upon adding a stop solution. The Avidin-Biotin-Peroxidase Complex (ABC) was used to amplify the signal. The concentration of TNF-α was determined by measuring the optical density of each well using a UV/visible spectrophotometer at a specific wavelength^21,22^.

### Interleukin 6 (IL-6) level determination

The manufacturer’s instructions were followed to assess the concentration of IL-6 in rat serum using rat IL-6 ELISA kit and monoclonal anti-rat antibody for IL-6 (Sunlong Biotechnology, Hangzhou, Zhejiang, China). The absorbance is proportional to the quantity of rat IL-6 collected on plate^42^.

### Histological examination

The intestinal segments and the surrounding adhesion tissues were carefully dissected. The harvested samples were fixed in a 10% neutral buffered formalin for 24 h. Subsequently, all tissue specimens underwent standard processing for histopathological evaluations. Thin section (5 μm) were prepared and stained with hematoxylin and eosin (H&E) for microscopic examination using a light microscope (CX31; Olympus, Tokyo Japan). Images were captured with a digital camera (Toupview, LCMos10000KPA, China) in the Photomicrograph Laboratory, Department of Pathology, Faculty of Veterinary Medicine, Assiut University. Histopathological evaluation was done blindly on samples. According to Table 2 as reported previously, the degree of inflammation was measured on a scale of 0 to 3^6^.

**Table 2:** The* in vivo* degree of inflammation following the criteria described by Wu et al.^6^.

Degree of inflammation	
Scale	Inflammation
0	No inflammation.
1	Presence of giant cell, occasional lymphocytes, and plasma cell.
2	Presence of giant cell, plasma cell, eosinophils, and neutrophils.
3	Presence of many inflammatory cells and microabscess formation.

### Immunohistochemical assessment of macrophages

For immunohistochemical (IHC) analysis of macrophages, paraffin-embedded sections from the cecum and adhesion regions (collected 2 weeks post-surgery) were used for detection of Anti-CD163 antibody [EPR19518] (ab182422, Abcam, Cambridge, UK) at a dilution of 1:200. The tissue section (3 μm thick) were deparaffinized and rehydrated in a descending series of alcohols. Following the antibody manufacturer’s protocol, heat-induced antigen retrieval was performed using a microwave and citrate buffer (pH 6) for 20 min. Endogenous peroxidase activity was blocked with 3% hydrogen peroxide. The slides were then incubated overnight in the primary antibody (diluted in phosphate-buffered saline) at 4 ^◦^C in a humidified chamber. Next, the sections were incubated with Econo Tek biotinylated anti-polyvalent for 30 min at room temperature. The tissues were washed four times with phosphate-buffered saline, each for 5 min, followed by incubation with Econo Tek HRP Conjugate for 30 min. To visualize the staining, a DAB chromogen mixture was applied, and the sections were incubated with the DAB substrate for 10 min. Finally, the sections underwent hydration, hematoxylin counterstaining, dehydration, and mounting for analysis. The positive immunoreactions appeared as a brown coloration. Microphotographs from five random fields (40X) for the semi-quantitative analysis were taken. The percentage of positively stained areas was used to score Anti-CD163 antibody immunostaining on a semi-quantitative scale^43^. The following criteria were used to grade the Anti-CD163 antibody staining intensity. The percentage of cells exhibiting positive staining was classified into five categories: 0 = 0%, 1 = 1–25%, 2 = 26–50%, 3 = 51–75%, and 4 = 76–100%. The mean value for each group represented the final staining score.

### Statistical analysis

Data from *in vitro* cytotoxicity (*n* = 8), gross adhesion score (*n* = 6), biochemical parameters (*n* = 6), degree of inflammation (*n* = 6), and IHC macrophage staining (*n* = 6) were presented as mean ± SD. Statistical analysis was conducted using one-way analysis of variance (ANOVA) followed by Tukey’s post hoc test with a *p*-value of < 0.05 was considered significant. IBM SPSS® Statistics (Version 21, USA) was used to operate statistical analysis.

## Results

### Tadalafil/cellulose composite characterization

**Fig. 2:**
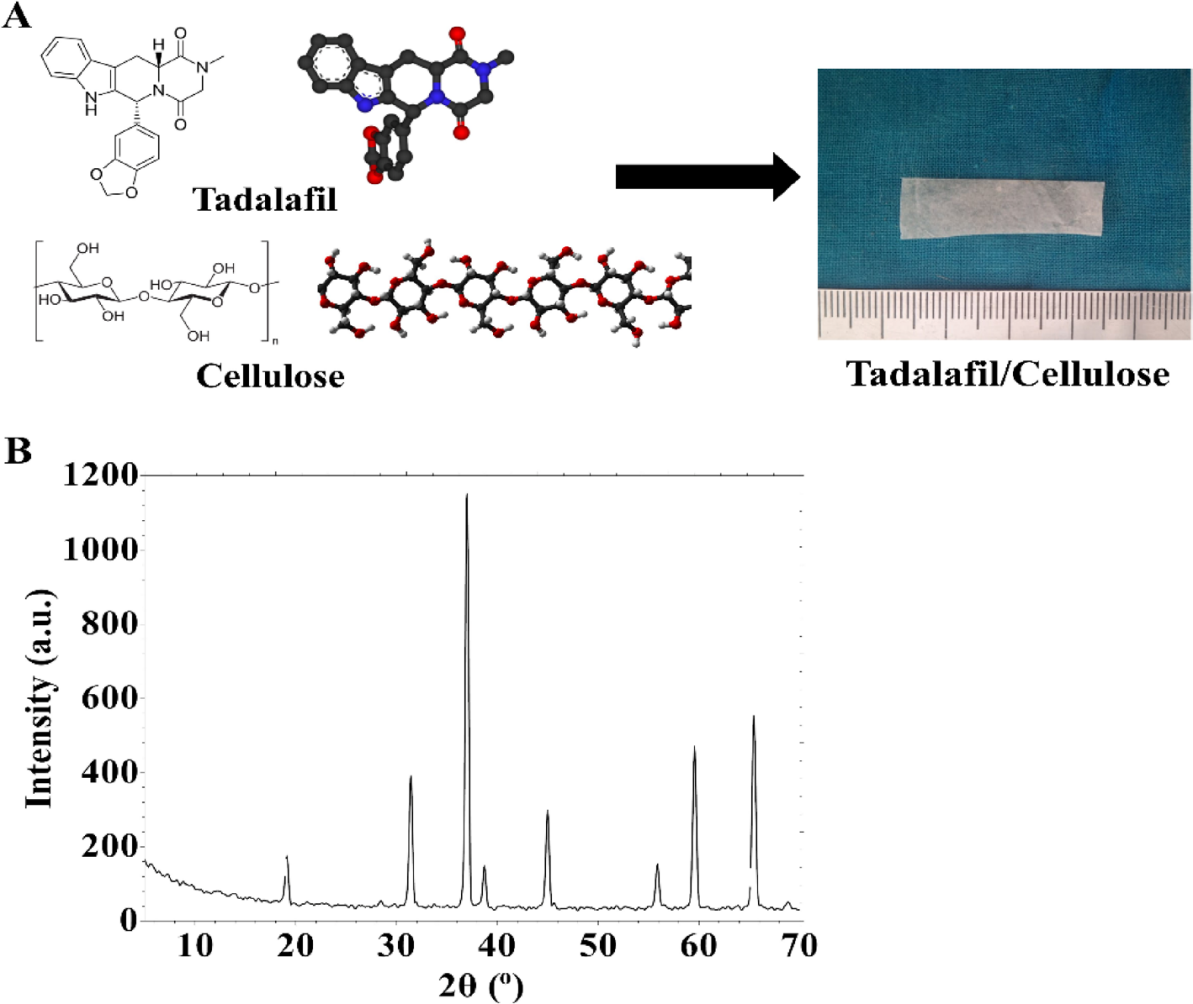
Characterization of tadalafil/cellulose. (**A**) Preparation procedure and (**B**) XRD characterization of tadalafil/cellulose.

Figure 2A shows the chemical structure of tadalafil and cellulose. It also demonstrates thin film preparation with tadalafil/cellulose via casting. The synthesis procedure offers a thin white film (Fig. 2A). XRD diffraction of the prepared thin film showed sharp diffraction peaks corresponding to the tadalafil drug in the prepared thin film (Fig. 2B). The interaction between the drug and cellulose is mainly electrostatic and hydrogen bond forces^44,45^.

### *In vitro* cytotoxicity assay

**Fig. 3:**
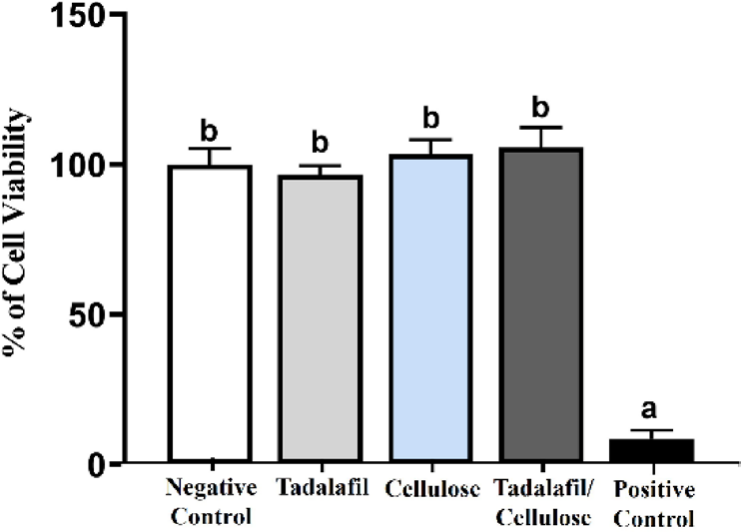
Cytotoxicity assay *in vitro*. Cell viability of mouse embryonic fibroblast cells determined by the MTT assay. Cells were treated with tadalafil, cellulose, or a combination of tadalafil and cellulose for 48 h. Each bar represents the mean ± SD of cell viability percentage from three independent experiments (*n* = 8). The groups that labeled with different letters were statistically significant (*p* < 0.05), while groups having the same letter were insignificant. Differences were evaluated using one-way ANOVA followed by Tukey’s HSD post hoc test.

The negative control group, representing untreated cells, was set at 100% viability. Cells treated with tadalafil (96.4 ± 3.1%), cellulose (103.5 ± 4.7%), or the tadalafil/cellulose combination (105.5 ± 6.81%) exhibited no significant effect on viability compared to the negative control (Fig. 3). In contrast, the positive control group treated with 20% DMSO showed a significant decrease in cell viability (8.5 ± 2.95%), confirming the assay’s ability to detect cytotoxic effects. These findings indicate that tadalafil, cellulose, and their combination are non-cytotoxic at the tested concentrations.

### Gross findings

**Fig. 4:**
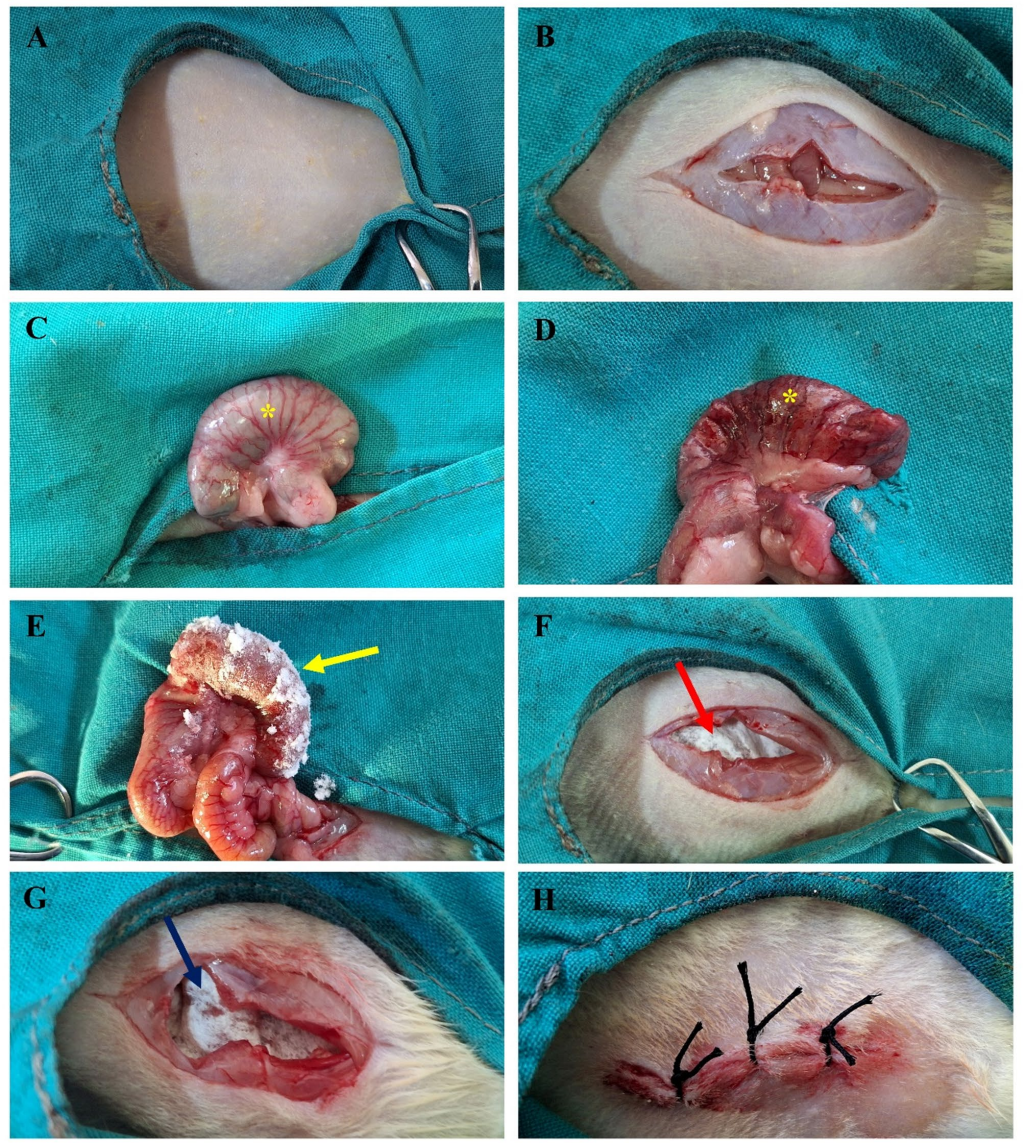
Surgical establishment of cecal abrasion model. (**A**) Strict aseptic preparation, (**B**) celiotomy, (**C**) cecal exteriorization (yellow asterisk), (**D**) induction of cecal abrasion (yellow asterisk), (**E**) local application of tadalafil powder on the abraded cecum, (**F**) application of a cellulose sheet (red arrow), (**G**) application of a tadalafil/cellulose sheet (dark blue arrow), and (**H**) abdominal wall suturing.

**Fig. 5:**
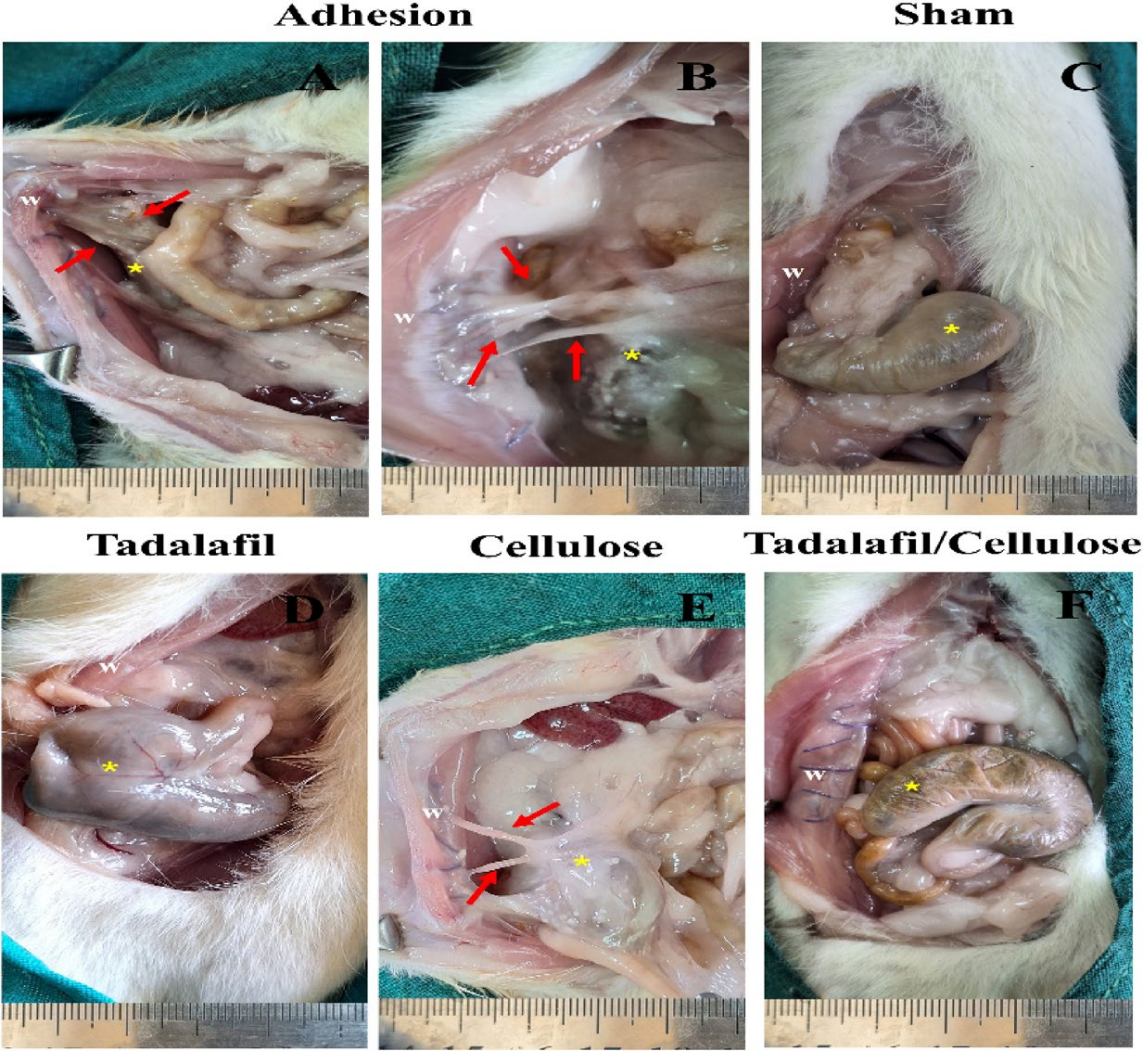
Gross appearance findings of intra-abdominal adhesion formation. The adhesion group showed severe intra-abdominal adhesions (**A**, **B**). The sham group displayed no abdominal adhesions (**C**). The gross appearance in tadalafil-treated group revealed no abdominal adhesions formation (**D**). The cellulose-treated group showed moderate abdominal adhesions (**E**). No adhesions were observed in the tadalafil/cellulose-treated group (**F**). W = abdominal wall, yellow asterisk: cecum, red arrow: adhesion bands.

After the induction of cecal abrasions in rats as shown in Fig. 4, the intra-abdominal adhesions formation was evaluated grossly after 2 weeks. Rats in the sham group exhibited no adhesions in the entire abdomen, whereas the adhesion group displayed the presence of dense bands of adhesions between the abraded cecum, adjacent intestinal segments, and abdominal wall, indicating severe intra-abdominal adhesions formation (grade 2 and 3) (Fig. 5).

In the tadalafil-treated group, intra-abdominal adhesion formation was absent in all rats except for one, which revealed the presence of a delicate thin adhesion band of grade 1 between the wall of the abdomen and cecum. In contrast, the cellulose-treated rats showed intra-abdominal formation of adhesions (grade 2 and 1) between the abdominal wall and intestinal loops with the cecal serosa. However, no adhesions were observed intra-abdominally in tadalafil/cellulose-treated group. Notably, the cellulose and tadalafil/cellulose sheets were undetectable upon gross examination, indicating their complete resorption.

Regarding adhesion scoring (Table 3), the different treated groups showed a significant reduction in adhesion formation compared to the adhesion group (*p* < 0.0001 for cellulose group, and *p* < 0. 00001 for other treated groups). However, no significant difference was found between the sham group and the treated groups, except for the cellulose-treated group (*p* < 0.0001). Additionally, the cellulose-treated group exhibited a significantly higher gross adhesion score compared to both the tadalafil- and tadalafil/cellulose-treated groups (*p* < 0.001 and *p* < 0.0001, respectively).

**Table 3:** The gross findings of the *in vivo* adhesion scoring.

	Adhesion group	Tadalafil group	Cellulose group	Tadalafil/ Cellulose group	Sham group
	Mean ± S.D.	Mean ± S.D.	Mean ± S.D.	Mean ± S.D.	Mean ± S.D.
Adhesion degree score	2.66 ± 0.51 c	0.16 ± 0.40 a	1.33 ± 0.52 b	0.00 ± 0.00 a	0.00 ± 0.00 a

The groups that labeled with different letters were statistically significant (*p* < 0.05), while groups having the same letter were insignificant. Differences were evaluated using one-way ANOVA followed by Tukey’s HSD post hoc test.

### Oxidative stress indicators outcome

**Fig. 6:**
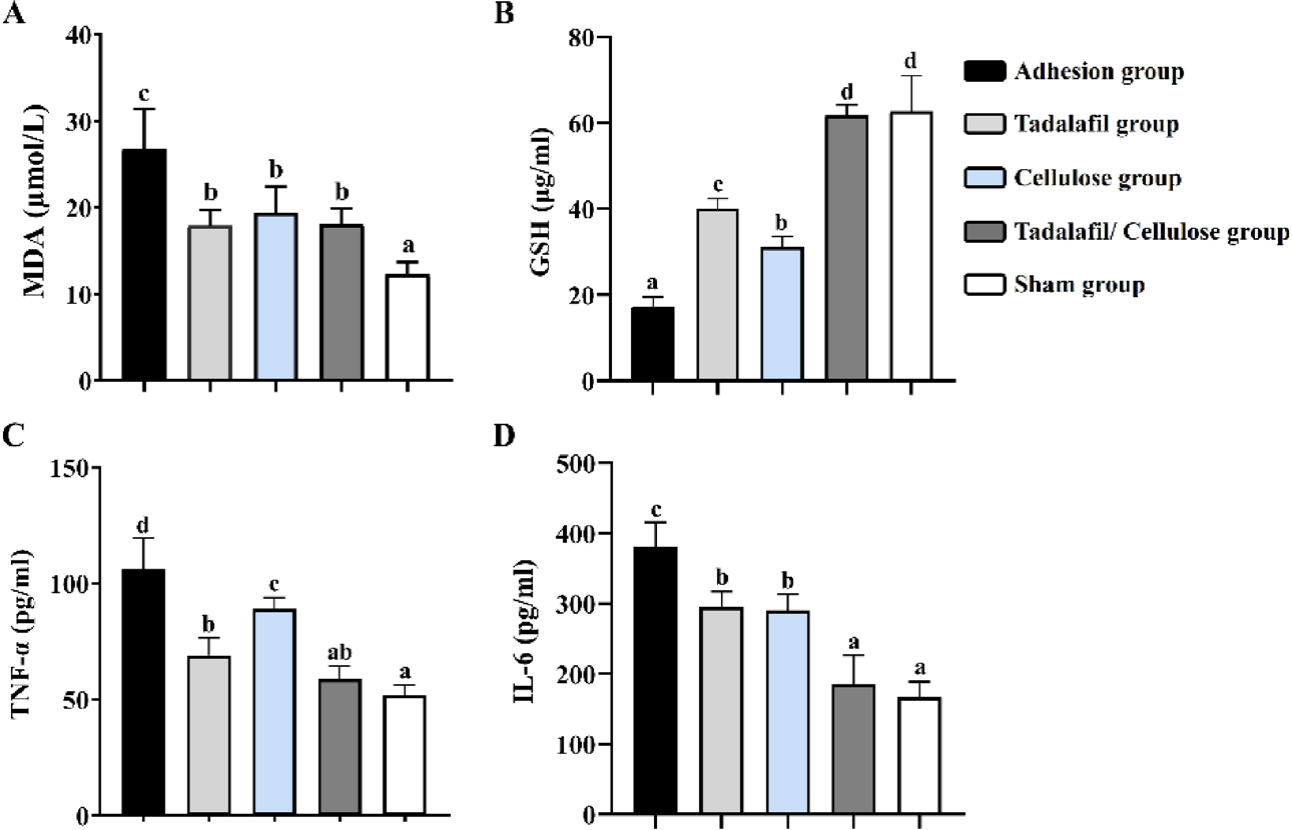
Biochemical indicators of the adhesion formation. (**A**) MDA (µmol/L), (**B**) GSH (µg/mL), (**C**) TNF-α (pg/mL), and (**D**) IL-6(pg/mL). The groups that labeled with different letters were statistically significant (*p* < 0.05), while groups having the same letter were insignificant. Differences were evaluated using one-way ANOVA followed by Tukey’s HSD post hoc test.

As presented in Fig. 6A, serum MDA levels at week 2 were highest in the adhesion group (26.75 ± 4.59 µmol/L) compared to all other study groups (*p* < 0.0001 for tadalafil, *p* < 0.01 for cellulose group, *p* < 0.001 for tadalafil/cellulose, and *p* < 0.00001 for sham groups). The MDA levels in the sham group (12.20 ± 1.48 µmol/L) was significantly lower than those in tadalafil (17.86 ± 1.88 µmol/L; *p* < 0.05), cellulose (19.40 ± 3.04 µmol/L; *p* < 0.01), and tadalafil/cellulose (18.08 ± 1.80 µmol/L; *p* < 0.05) groups.

Conversely, the level of GSH in serum was lowest in the adhesion group (17.21 ± 2.28 µg/mL) (*p* < 0.0001 for cellulose group, and *p* < 0.00001 for tadalafil, tadalafil/cellulose, and sham groups) (Fig. 6B). However, the sham and tadalafil/cellulose groups (62.69 ± 8.25 and 61.74 ± 2.49 µg/mL, respectively) had significantly higher GSH levels than both the tadalafil (40.06 ± 2.34 µg/mL; *p* < 0.00001) and cellulose (31.08 ± 2.53 µg/mL; *p* < 0.00001) groups. Additionally, the tadalafil-treated group showed significantly higher GSH levels than the cellulose-treated group *(p* < 0.05).

### TNF-α and IL-6 levels

In the sham group, a significant decrease compared to the adhesion, tadalafil treated, and cellulose treated groups was observed in the levels of TNF-α (51.95 ± 4.21 pg/mL; *p* < 0.01 for tadalafil group and *p* < 0.00001 for adhesion and cellulose groups) and IL-6 (166.53 ± 22.27 pg/mL; *p* < 0.00001 for all groups). Conversely, the serum levels of TNF-α (106.07 ± 13.43 pg/mL) in the adhesion group showed a significant increase compared to all treated groups (*p* < 0.01 for cellulose group, *p* < 0.00001 for tadalafil and tadalafil/cellulose-treated group). In addition, a higher level of IL-6 in the adhesion group (380.09 ± 35.15 pg/mL) was reported compared to the tadalafil (*p* < 0.001), cellulose (*p* < 0.001), and tadalafil/cellulose treated groups (*p* < 0.00001) (Fig. 6C and D).

Regarding TNF-α levels, the cellulose-treated rats displayed a higher level (89.05 ± 4.86 pg/mL) relative to both the tadalafil- (69.11 ± 7.50 pg/mL; *p* < 0.01) and tadalafil/cellulose- (58.73 ± 5.71 pg/mL; *p* < 0.00001) treated rats. However, no significant difference was observed in IL-6 levels between tadalafil- and cellulose-treated groups. The IL-6 levels in the tadalafil/cellulose-treated group (185.56 ± 40.78 pg/mL) were significantly lower than in the tadalafil- and cellulose-treated groups (294.94 ± 22.18; *p* < 0.0001 and 290.38 ± 22.98 pg/mL; *p* < 0.00001, respectively).

### Histopathological findings

**Fig. 7:**
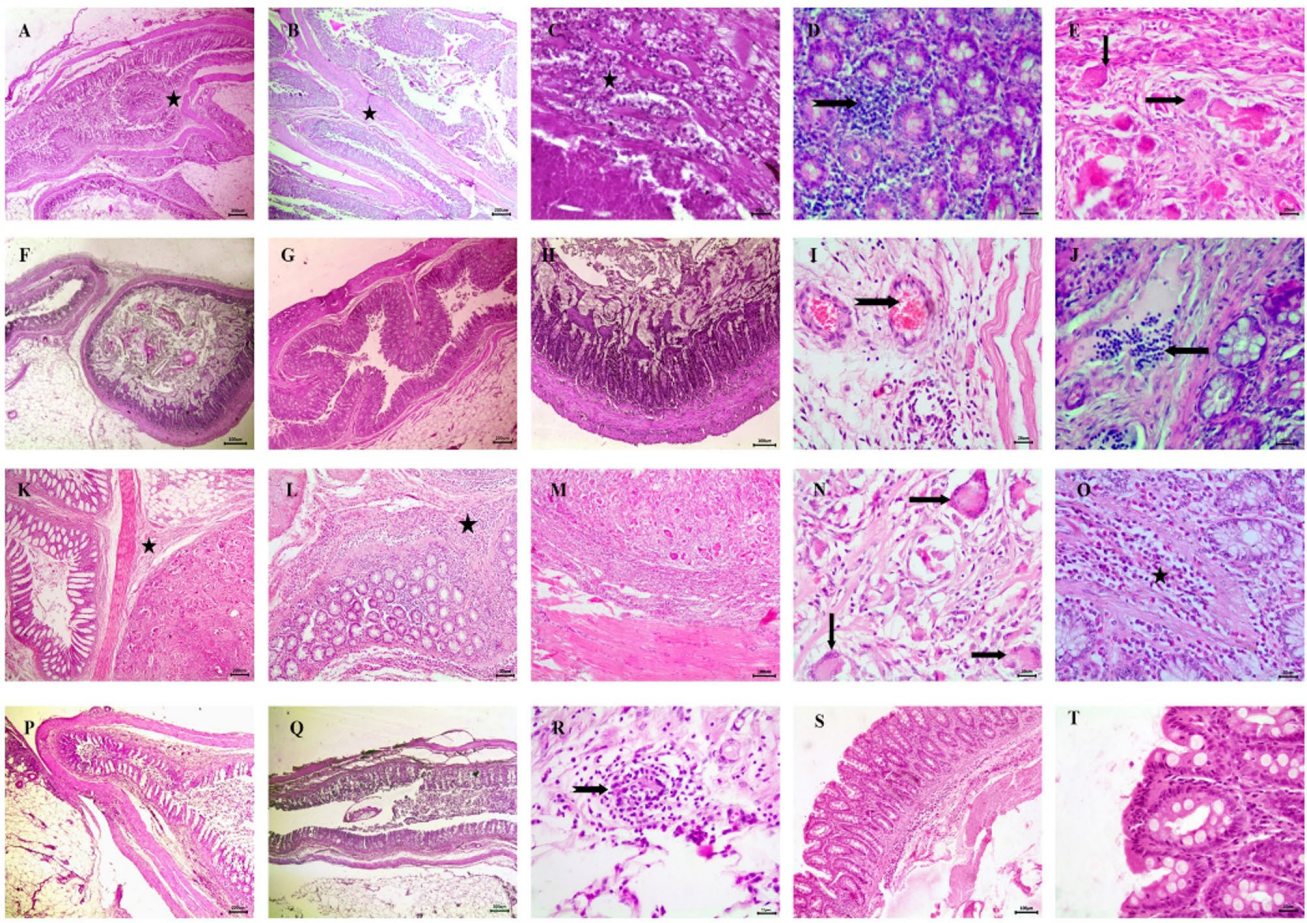
Histopathological examination of intestine and adhesion tissue by H&E staining. The adhesion group displayed (**A**, **B**) an adhesion site between intestines (star), and (**C**, **D**, **E**) infiltration of neutrophils (star), infiltration between mucosal intestinal glands with mononuclear inflammatory cells and giant cells (arrows). The tadalafil-treated group showed (**F**, **G**, **H**) no adhesion, and (**I**, **J**) hyperemia of blood vessels (notched arrow) with mild mucosal inflammatory cell infiltration (arrow). The cellulose-treated group showed (**K**, **L**) an adhesion site between intestines (star), and (**M**, **N**, **O**) giant cells (arrows), mucosal inflammatory cellular infiltration (star). The tadalafil/cellulose-treated group displayed (**P**, **Q**) no adhesion, while (R) showed giant cells (notched arrow). The sham group (**S**) showed no adhesion and (**T**) normal intestinal mucosa. The scale bars in panels (A, B, G, K, L, P, and Q) = 200 μm, panels (F, H, M, and S) = 100 μm, (C, D, E, I, J, N, O, R, and T) = 20 μm.

Histopathological examination of the intestine and adhesion tissue in the adhesion group showed severe, extensive adhesions between intestinal segments (Fig. 7A, B). The adhesion tissues and intestine showed marked infiltration with plenty of inflammatory cells, including mononuclear inflammatory cells, giant cells, and neutrophils (Fig. 7C–E). In the tadalafil-treated group, no adhesions were observed (Fig. 7F–H) associated with inflammatory reaction in some segments characterized by hyperemia of submucosal blood vessels and mild mucosal inflammatory cellular infiltration between intestinal glands (Fig. 7I, J). However, the cellulose-treated group displayed mild adhesions between intestinal segments (Fig. 7K, L), with inflammatory cellular reaction in the intestinal serosa and parietal peritoneum, consisting of various inflammatory cells and giant cells (Fig. 7M–O). In contrast, no adhesions were detected in the tadalafil/cellulose-treated group (Fig. 7P, Q), although mild mononuclear inflammatory cell infiltration was observed in the intestinal serosa (Fig. 7R). The sham group exhibited normal intestinal morphology without adhesions or inflammatory reaction (Fig. 7S, T).

As presented in Table 4, the histopathological scoring of the degree of inflammation revealed significantly higher inflammatory reactions in the adhesion group compared to the tadalafil- (*p* < 0.00001), cellulose-(*p* < 0.05), and tadalafil/cellulose-treated groups (*p* < 0.00001), as well as the sham group (*p* < 0.00001). Additionally, the cellulose-treated group exhibited significantly higher inflammation scores than the tadalafil (*p* < 0.001), tadalafil/cellulose (*p* < 0.01), and sham groups (*p* < 0.00001).

**Table 4:** The score of the *in vivo* degree of inflammation and the intensity of macrophage expression in IHC staining.

	Adhesion group	Tadalafil group	Cellulose group	Tadalafil/ Cellulose group	Sham group
	Mean ± S.D.	Mean ± S.D.	Mean ± S.D.	Mean ± S.D.	Mean ± S.D.
Inflammation degree score	2.10 ± 0.56 c	0.40 ± 0.51 a	1.40 ± 0.69 b	0.50 ± 0.52 a	0.00 ± 0.00 a
Macrophage IHC score	3.10 ± 0.73 d	1.10 ± 0.56 b	1.90 ± 0.73 c	1.00 ± 0.66 b	0.00 ± 0.00 a

The groups that labeled with different letters were statistically significant (*p* < 0.05), while groups having the same letter were insignificant. Differences were evaluated using one-way ANOVA followed by Tukey’s HSD post hoc test.

### IHC expression of macrophages

**Fig. 8:**
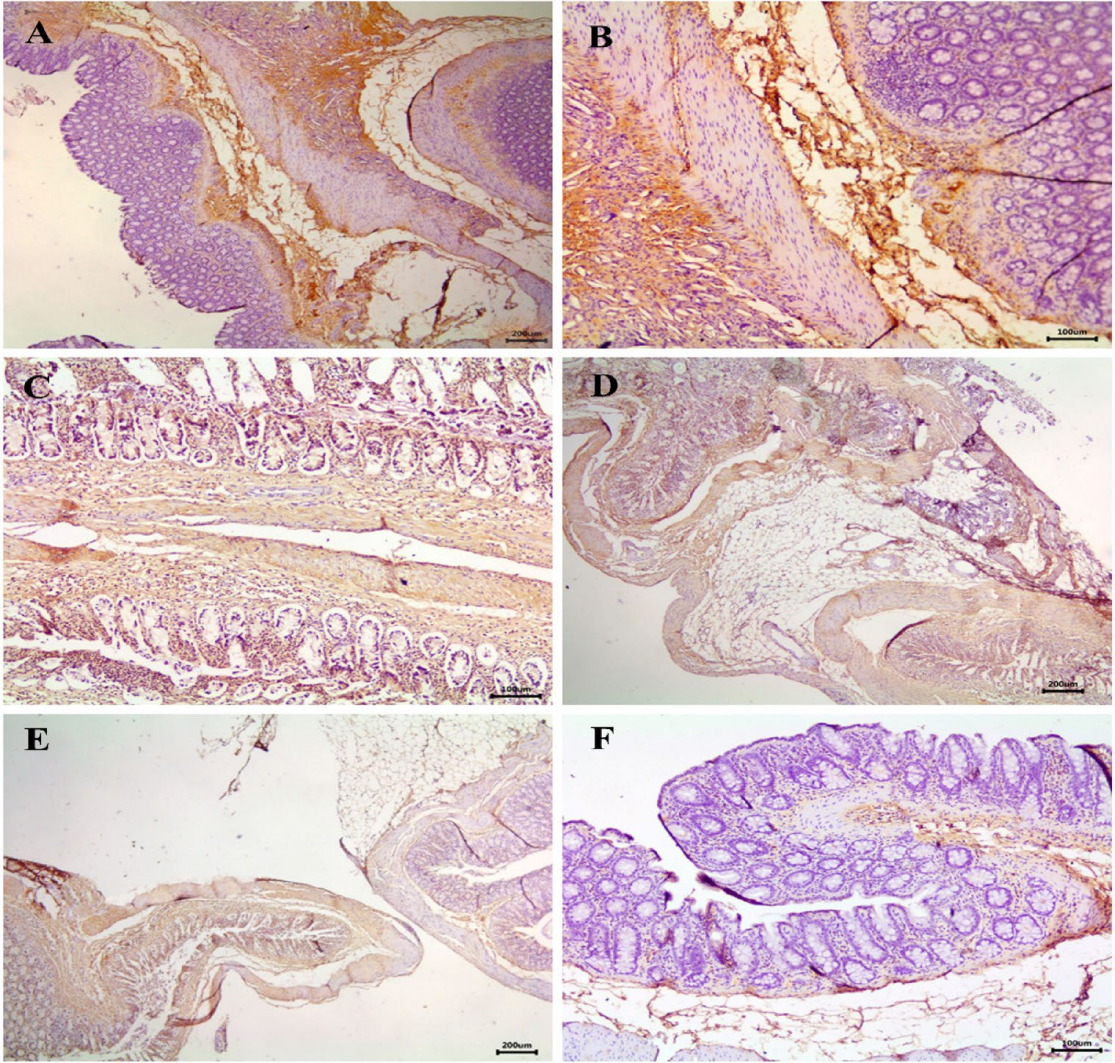
Immunohistochemical staining for macrophage marker (Anti-CD163 antibody) in the intestine and adhesion site. (**A**, **B**) The adhesion group showed severe positive reaction with a severe density for macrophages. (**C**) The tadalafil-treated group, (**D**) The cellulose-treated group, and (**E**) the tadalafil/cellulose-treated group displayed no adhesion but showed a moderate positive reaction with moderate macrophage density (brownish cytoplasmic stain). (**F**) The sham group revealed no reaction. The scale bars in panels (A, D, and E) = 200 μm, panels (B, C, and F) = 100 μm.

IHC staining for macrophage in the adhesion group showed intense positive staining, indicating severe macrophage infiltration (Fig. 8A, B). In contrast, no staining was observed in the sham group (Fig. 8F). Among the treated groups, the tadalafil-treated group displayed mild macrophage infiltration (Fig. 8C), while the cellulose-treated group showed moderate macrophage infiltration (Fig. 8D). The tadalafil/cellulose-treated group displayed weak positive reaction (Fig. 8E).

IHC scoring (Table 4) revealed that the adhesion group had the highest macrophage staining intensity (3.10 ± 0.73). The sham group had the lowest macrophage expression, significantly lower than both the tadalafil- and cellulose-treated groups (*p* < 0. 0.01 and *p* < 0.00001, respectively). Furthermore, the tadalafil- and tadalafil/cellulose-treated groups exhibited significantly lower macrophage expressions compared to the cellulose-treated group (1.10 ± 0.56, 1.00 ± 0.66, and 1.90 ± 0.73, respectively; *p* < 0.05).

## Discussion

The intra-abdominal adhesion formation is a challenging postoperative problem that faces the surgeons, leading to serious health and financial complications. Consequently, preventing these intra-abdominal adhesions is crucial and can be achieved through optimizing surgical conditions, including strict aseptic techniques, the use of minimally traumatic surgical approaches and powder-free gloves, and the physical separation of adhesion-prone surfaces^10,11,38^. In addition, the application of anti-adhesive substances is beneficial in preventing postoperative adhesion. These anti-adhesive materials must be biocompatible, biodegradable, easy to apply, and capable of completely covering the injured area while preventing contact with surrounding tissues during the critical period of adhesion formation^7,9^. Despite extensive research on various therapeutic agents, no ideal solution for preventing intra-abdominal adhesions has been established for clinical use, with outcomes remaining inconsistent^10^. This study evaluates the potential of application of tadalafil, cellulose, or tadalafil/cellulose in preventing or alleviating postsurgical intra-abdominal adhesion in a rat cecal abrasion model.

The suitability of materials for biomedical applications largely depends on their biocompatibility^46^. In this study, the MTT assay demonstrated that tadalafil, cellulose, and their combination did not exhibit cytotoxic effects on mouse embryonic fibroblast cells, as cell viability remained comparable to the negative control. The absence of significant cytotoxicity suggests that these materials are well tolerated by cells and may support cellular functions. This finding aligns with previous studies reporting the biocompatibility of cellulose-based biomaterials and tadalafil in tissue engineering applications^47–50^. The results further support the potential use of these materials in biomedical applications without adverse effects on cell viability.

The process of adhesion formation involves complex overlapping interactions that are evoked following serosal or peritoneal surfaces trauma during surgical manipulation of abdominal organs. This serosal or peritoneal surfaces injury triggers local tissue hypoxia and reactive oxygen species (ROS) release as well as increased vascular permeability, leading to an exaggerated inflammatory response. The influx of inflammatory cells, including neutrophils, monocytes, macrophages, and lymphocytes, stimulates cytokine production, such as TNF-α, IL-1, IL-6, platelet-derived growth factor (PDGF), and transforming growth factor-β (TGF-β). These cytokines downregulate fibrinolysis, promoting the formation of a fibrin-rich matrix on the damaged serosa, which subsequently attracts fibroblasts and leads to vascularization and excessive collagen deposition, ultimately resulting in firm adhesion formation^7,9–11^.

In this study, macroscopic and microscopic findings confirmed that local application of tadalafil, cellulose, or tadalafil/cellulose significantly reduced intra-abdominal adhesion formation compared to the untreated adhesion group. Notably, tadalafil/cellulose demonstrated the most effective prevention, completely inhibiting adhesion formation. In the tadalafil-treated group, adhesions were undetectable between the injured serosa and adjacent tissues, with no collagen deposition. Although one rat in the tadalafil-treated group exhibited a thin adhesion band during gross evaluation, which was included in the gross adhesion score, no corresponding adhesions were observed in the histopathological sections. This discrepancy may be attributed to the possible loss or dislocation of the delicate adhesion band during tissue processing and sectioning. This effect may be attributed to the inhibitory effect of tadalafil on the PDE-5 activity that is associated with an elevation of cyclic guanosine monophosphate (cGMP) level associated with vasodilatation, reduction of inflammatory response and collagen fiber deposition^22,27^. In contrast, cellulose applications formed mild adhesions with immature collagen deposition. The reduction in adhesion severity is likely due to cellulose’s ability to act as a degradable physical barrier that isolates injured tissue from surrounding structures and enhances fibrinolysis by upregulating tissue-type plasminogen activator (tPA) and increasing the tPA/plasminogen activator inhibitor-1 (tPA/PAI-1) ratio^9,51,52^. The tadalafil/cellulose-treated group completely prevented adhesion formation, suggesting a synergistic effect between tadalafil and cellulose, where tadalafil enhances the anti-adhesive properties of cellulose.

Oxidative stress plays a key role in postoperative adhesion formation. After surgery, elevated ROS levels and impaired antioxidant defenses predispose peritoneal mesothelial cells to damage, triggering an exaggerated inflammatory response that promotes adhesion development^6^. Herein, the levels of MDA and GSH levels were assessed as oxidative stress and antioxidant markers, respectively, to evaluate the treatment effects on the oxidative/antioxidative balance to prevent postoperative adhesion. Our results showed that the level of MDA in the treated groups was significantly reduced compared to the adhesion group. In contrast, the GSH level was increased in the treated groups relative to the adhesion group, suggesting that the different treatments exhibited an antioxidant activity. The antioxidant effect of tadalafil may be linked to PDE-5 inhibition, which elevates cGMP levels and reduces nicotinamide adenine dinucleotide phosphate (NADPH) oxidase activity, leading to vasodilation, enhanced cellular antioxidation, and reduced ROS-induced damage^21,22,53^. While the antioxidant activity of cellulose is suggested to be attributed to its ability to stimulate antioxidant system as well as reduce the ROS metabolism and prevent membrane lipid peroxidation^54^.

Interestingly, the potential of tadalafil, cellulose, and tadalafil/cellulose to break down the process of adhesion formation was confirmed by measuring TNF-α and IL-6 level as a reliable pro-inflammatory markers^55^. These cytokines suppress fibrinolysis by downregulating tPA, thereby decreasing the tPA/PAI-1 ratio and fibrinolytic activity^1,11^. Our data show that tadalafil, cellulose, and tadalafil/cellulose considerably reduced the level of both TNF-α and IL-6 compared to those of the adhesion group. These results suggest that the potential of the designed materials to reduce or prevent the adhesions formation are related to the anti-inflammatory feature of tadalafil and cellulose^21,56^. These findings are consistent with previous reports demonstrating the anti-inflammatory properties of tadalafil^57–59^ or cellulose^60,61^.

The reduction in oxidative stress and pro-inflammatory cytokines correlated with a significant histopathological decrease in the inflammatory response in treated groups compared to the adhesion group. Another notable finding was the suppression effect in the tadalafil, cellulose, and tadalafil groups on the recruitment of macrophages, as evidenced by reduced Anti-CD163 antibody expression. These findings proved the capability of the different treatment materials in alleviating or preventing the development of post-surgical abdominal adhesions because macrophages, the predominant cells at adhesion tissue, are key players in adhesion formation, as they secrete cytokines, growth factors, and ROS that drive inflammation and tissue fibrosis^11^. The observed reduction in macrophage infiltration suggests an immunomodulatory effect of tadalafil^62^ and cellulose^63^. This immunomodulatory effect of tadalafil may be attributed to PDE-5 inhibition, which results in downregulation of pro-inflammatory markers and reduced macrophage recruitment. Elevated cGMP levels, resulting from PDE-5 inhibition, have been shown to decrease monocyte adhesion to endothelial cells, thereby limiting their transmigration and subsequent differentiation into macrophages^64^.

In conclusion, tadalafil, cellulose, and tadalafil/cellulose are biodegradable and biocompatible materials with an *in vivo* potential to inhibit the formation of the intra-abdominal adhesions postoperatively. Among these, tadalafil/cellulose demonstrated the most effective prevention. This protective effect of these materials is attributed to their antioxidation and anti-inflammatory behavior of these materials, as confirmed by their regulatory effect on oxidation/antioxidation markers, pro-inflammatory markers, collagen deposition, and macrophage recruitment. Hence, tadalafil and tadalafil/cellulose appear to be promising candidates for preventing postsurgical peritoneal adhesion formation.

Future studies should explore these materials over long-term duration, using larger animal models and varied adhesion induction protocols. Further research should also evaluate their impact on fibrinolytic activity and confirm the precise mechanisms underlying their anti-adhesive effects.

## Data Availability

The data that support the findings of this study are available from the corresponding author upon reasonable request.

## Abbreviations

cGMP: Cyclic guanosine monophosphate
DMSO: Dimethyl sulfoxide
ELISA: Enzyme-linked immunosorbent assay
FBS: Fetal bovine serum
H&E: Hematoxylin and eosin
IHC: Immunohistochemistry
IL-6: Interleukin-6
MDA: Malondialdehyde
MEF: Mouse embryonic fibroblast
MTT: [3-(4,5-dimethylthiazol)-2-yl]-2,5- diphenyltetrazolium bromide
NADPH: Nicotinamide adenine dinucleotide phosphate
PDE-5: Phosphodiesterase-5
PDGF: Platelet-derived growth factor
ROS: Reactive oxygen species
GSH: Reduced glutathione
ABC: Avidin-biotin–peroxidase complex
tPA: Tissue-type plasminogen activator
tPA/PAI-1: Tissue-type plasminogen activator/plasminogen activator inhibitor-1
TGF-β: Transforming growth factor-β
TNF-α: Tumor necrosis factor-alpha
XRD: X-ray diffraction
